# CD36 enhances sensitivity of triple negative breast cancer cells to palmitate-induced ferroptosis

**DOI:** 10.1038/s41419-026-08460-3

**Published:** 2026-02-11

**Authors:** Lara Closset, Jean-Philippe Foy, Lila Louadj, Elodie Pramil, Elisabetta Marangoni, Ivan Bieche, Michèle Sabbah

**Affiliations:** 1 Centre de Recherche Saint-Antoine, CRSA, Sorbonne Université, Inserm, Paris, France; 2 Department of Maxillo-Facial Surgery, Hôpital Pitié-Salpêtrière, Assistance Publique des Hôpitaux de Paris, Paris, France; 3 Alliance for Research in Cancerology-APREC, Tenon Hospital, Paris, France; 4 Laboratory of Preclinical Investigation, Translational Research Department, Institut Curie, PSL University, Paris, France; 5 Department of Genetics, Institut Curie, Paris, France; 6 Faculty of Pharmaceutical and Biological Sciences, Paris City University, Inserm U1016, Paris, France; 7 Centre National de la Recherche Scientifique (CNRS), Paris, France

**Keywords:** Breast cancer, Cell death

## Abstract

Ferroptosis is a newly identified programmed cell death induced by iron-driven lipid peroxidation and implicated as a potential approach for tumor treatment. Breast tumors develop in a complex microenvironment whose main component is adipose tissue and gain aggressiveness through increased fatty acid uptake. Here, we demonstrated that palmitic acid (PA) induced ferroptosis in triple negative breast cancers (TNBC). We found that PA increases the protein expression levels of the long-chain fatty acid transporter CD36, leading to increased lipid uptake. Mechanistically, overexpression of CD36 increases lipid peroxidation, mitochondrial ROS production, the labile iron pool, and especially Fe^2+^ content. Additionally, we found increased expression of ferroptotic target genes (HMOX1, ACSL1, SAT1) and decreased of anti-ferroptotic genes (GPX4 and FSP1) in TNBC following PA exposure. Overexpression of CD36 did not induce ferroptosis in estrogen receptor positive breast cancer. Clinically, higher CD36 expression correlated with the luminal androgen receptor (LAR) subtype of TNBC, known to exhibit a higher sensitivity to ferroptosis. Altogether, these data provide evidence for an essential role of the CD36 protein in the ferroptotic process induced by the saturated fatty acid PA, opening potential new therapeutic approaches promoting ferroptosis in the most aggressive breast cancers.

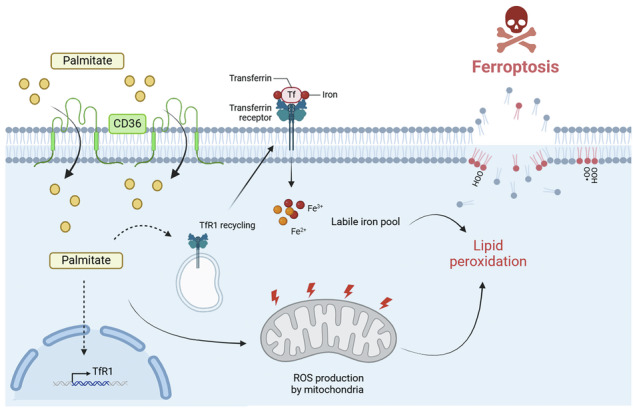

## Introduction

Breast cancer is the leading cancer in terms of incidence and mortality among women worldwide [1]. The advent of high-throughput sequencing techniques has conducted to the classification of breast cancer into different molecular subtypes corresponding to the luminal cancers expressing the estrogen and progesterone receptors (ER and PR, respectively), those over-expressing the HER2/Erb2 receptor (HER2), and the triple negative breast cancer (TNBC) characterized by the absence of expression of ER, PR, and HER2 [2]. Despite the fact that current treatments are able to target luminal and HER2 positive breast cancers, the main challenge remains for the most aggressive and resistant to anti-cancer therapies TNBC subtype.

Metabolic reprogramming of cancer cells is now recognized as a hallmark of cancer [3]. It is now well accepted that breast cancers undergo metabolic alterations leading to an increase uptake of free fatty acids coming from neighboring adipocytes [4]. The CD36 or fatty acid translocase protein is a membrane glycoprotein involved in the uptake of those long-chain fatty acids [5], and its ambivalent role in cancer progression and metastasis according to the metabolic context has been well documented [6, 7]. Indeed, recent studies have pointed out that fatty acids (FA) released by adipocytes induce the expression of CD36 in cancer cells, which increases the uptake of FA, leading to aggressiveness [8–13]. Palmitic acid (PA) is the most abundant saturated FA in breast tumor adipose tissue and is known to induce lipotoxicity. A newly described form of programmed cell death (PCD) related to lipid toxicity is ferroptosis [14]. Dixon et al. [14] first characterized this cell death by iron dependent lipid peroxidation leading to disrupt membrane properties and shrunken mitochondria. Ferroptosis is morphologically and mechanistically distinct from other PCD, such as apoptosis, necrosis, pyroptosis, and autophagy [15, 16]. Recent studies have shown that inducing ferroptosis could be an innovative strategy to target aggressive cancers, especially those with a drug- tolerant persister phenotype [17–23], but it remains challenging to identify biomarkers that could distinguish cancer cells that are inherently susceptible to ferroptosis [24].

However, little is known about the ability of PA to induce ferroptosis in TNBC cells. In this work, we demonstrated that PA is able to favor ferroptosis in TNBC but not in luminal breast cancer cells. Moreover, we showed that CD36 was able to amplify the ferroptotic process in TNBC by enhancing lipid peroxidation, mitochondrial reactive oxygen species accumulation, and iron overload through transferrin receptor 1 increased expression. By analyzing the expression of CD36 in patient-derived xenograft and in patient databases, we identified a higher CD36 expression in the luminal androgen receptor (LAR) subtype of TNBC. Overall, our results suggest that CD36 favors sensitivity of TNBC cells to PA-induced ferroptosis.

## Results

### Palmitic acid promotes cell death in different subtypes of breast cancer cells

**Fig. 1:**
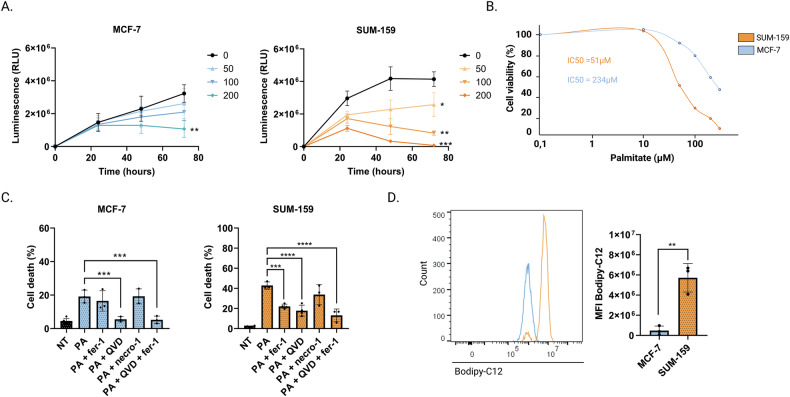
Palmitic acid promotes cell death in different subtypes of breast cancer cells. **A**, **B** MCF-7 and SUM-159 cells were treated with increasing doses of PA (50, 100 and 200 µM) during 24 h, 48 h, and 72 h. The half-inhibitory concentration (IC_50_) was evaluated after 48 h of treatment with PA. Cell viability was assessed by using ATP-based CellTiter-Glo ® 2.0 assay. **C** Cell death was determined by Annexin-V/7AAD co-labeling in MCF-7 and SUM-159 cells that were pre-incubated with fer-1 (5 µM), QVD (10 μM), or necro-1 (40 µM) for 6 h and then were treated with PA (200 μM) for 48 h. **D** PA uptake was evaluated in MCF-7 and SUM-159 cell lines by flow cytometry following staining with a PA fluorescent analog, the Bodipy FL-C12 (5 µM, 30 min). Data are expressed as a mean ± SD (*n* = 3). Data were analyzed by one-way ANOVA; ****p < 0.0001; ***p < 0.001; **p < 0.01; *p < 0.05 vs non-treated.

To study the effect of PA, we treated SUM-159 and MCF-7 breast cancer cells, corresponding to a TNBC and a luminal A breast cancer cells respectively, with increasing doses of PA (Fig. 1A). SUM-159 cells exhibited a higher sensitivity towards PA (IC_50_ = 51 ± 3,61 µM) compared to MCF-7 cells (IC_50_ = 234 ± 47,97 µM) in a dose and time dependent manner (Fig. 1A, B).

We found that PA was able to induce cell death in both MCF-7 and SUM-159 cells, as illustrated by the Annexin-V/7AAD staining (Fig. S1). To determine the types of cell death induced by PA, we treated MCF-7 and SUM-159 cells with Ferrostatin-1 (fer-1, ferroptosis inhibitor) and QVD.OPh (QVD, apoptosis inhibitor), and Necrostatin-1 (necro-1, necrosis inhibitor). As depicted in Fig. 1C, only the QVD was able to limit PA-induced cell death in MCF-7 cells, indicating an apoptotic process. Interestingly, in SUM-159 cells, QVD and fer-1 attenuated PA induced death, highlighting the sensitivity of SUM-159 cells to both apoptosis and ferroptosis. Those results were confirmed in two other luminal A breast cancer cells (ZR-75.1 and T47D) and two other TNBC cells (BT549 and MDA-MB-231) (Fig. S2A, B).

In order to understand those different vulnerabilities, we studied their ability to uptake PA by using the Bodipy FL-C12, a fluorescent FA analog to PA [25]. Interestingly, SUM-159 cells show a significantly increased uptake of Bodipy FL-C12 compared to MCF-7 cells (Fig. 1D), indicating that cell death observed in SUM-159 cells could be due to palmitate uptake.

### Lipid peroxidation, ROS, and iron accumulation are common mechanisms underlying palmitic acid induction of ferroptosis in TNBC cells

**Fig. 2:**
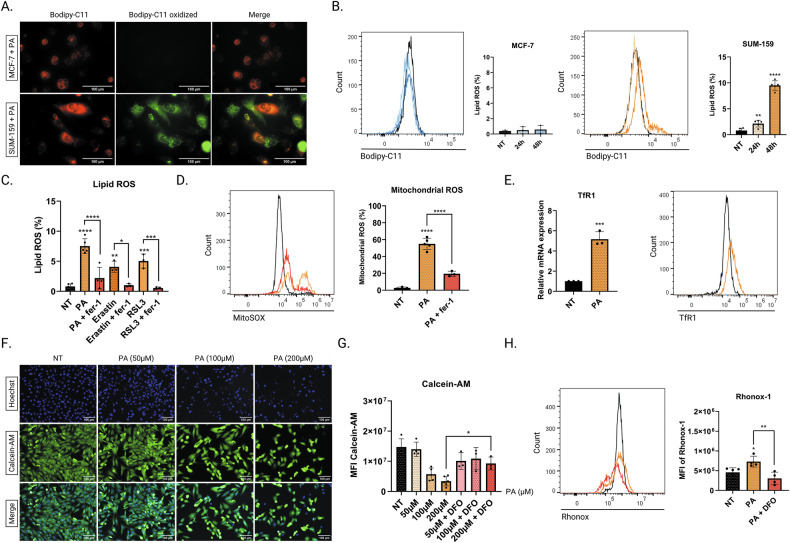
Lipid peroxidation, ROS and iron accumulation are common mechanisms underlying palmitic acid induction of ferroptosis in TNBC cells. The intracellular lipid ROS levels in MCF-7 and SUM-159 cells were analyzed with the Bodipy-C11 probe by (**A**) immunofluorescence after treatment with PA (200 µM, 24 h) or by (**B**) flow cytometry after treatment with PA (200 μM, 24 h and 48 h). **C** SUM-159 cells were treated with PA (200 µM), erastin-2 (80 µM) or with RSL3 (5 µM) alone for 48 h or after a pre-incubation with fer-1 (5 µM) for 6 h. The Bodipy-C11 oxidized form of the probe were quantified and expressed as a percentage. **D** Mitochondrial ROS was measured with MitoSOX (5 μM, 30 min) in SUM-159 cells that were treated with PA (200 µM, 48 h) alone or combined with fer-1 (5 µM). **E** mRNA and protein levels of TfR1 in SUM-159 cells treated with PA (200 µM, 24 h). **F** Iron labile pool was measured by calcein-AM (5 µM, 30 min) labelling of SUM-159 cells treated with PA (50 µM, 100 µM or 200 µM, 48 h) or **G** pre-incubated with DFO (100 µM, 6 h) before being treated with PA. **H** Ferrous iron (Fe^2+^) was evaluated by Rhonox-1 staining (5 µM, 30 min) in SUM-159 cells treated with PA (200 µM, 48 h) or pre-incubated with DFO (100 µM, 6 h) before being treated with PA. Data are expressed as a mean ± SD (*n* = 3). Data were analyzed by one-way ANOVA; ****p < 0.0001; ***p < 0.001; **p < 0.01; *p < 0.05 vs non treated.

We next studied different hallmarks of ferroptosis, such as lipid peroxidation, mitochondrial ROS production, and iron content. Our results revealed that PA treatment induces peroxidized lipid accumulation in SUM-159 cells, whereas MCF-7 cells showed no lipid peroxidation (Fig. 2A, B). Similar results were obtained with erastin-2 or RSL3, two well-known inducers of ferroptosis, while treatment with fer-1 significantly reduced lipid peroxidation, indicating that ferroptosis was implicated in this PA-induced process (Fig. 2C).

We then assessed the mitochondrial ROS accumulation following PA treatment of SUM-159 and MCF-7 cells stained with MitoSOX, a mitochondrial superoxide probe. PA-treated cells exhibited an increased mitochondrial ROS production, which was abrogated by fer-1, validating the important role of mitochondrial ROS in PA-induced ferroptosis only for SUM-159 cells (Figs. 2D and S3).

To functionally confirm that PA-treated SUM-159 cells undergo ferroptosis, we evaluated the expression of the transferrin receptor 1 (TfR1), which is responsible for iron delivery to cells. PA-treated SUM-159 cells exhibited increased mRNA and protein expression levels of TfR1 compared to their non-treated counterparts (Fig. 2E). Consistently, we assessed the labile iron pool (LIP) following PA treatment using calcein-AM fluorophore, which quenches in the presence of Fe^2+/3+^, and found that the LIP was increasing in the presence of PA in a dose-dependent manner. This phenomenon was abrogated by deferoxamine (DFO), an iron chelator, validating the role of PA in the elevation of the LIP in SUM-159 cells (Fig. 2F, G). Finally, we evaluated the concentration of ferrous iron (Fe^2+^) content by using Fe^2+^ specific turn-on fluorescent probe RhoNox-1. We found that PA increased Fe^2+^ level in SUM-159 cells and that combination of PA and DFO prevented this process (Fig. 2H).

### Palmitic acid enhances ferroptosis by upregulating CD36

**Fig. 3:**
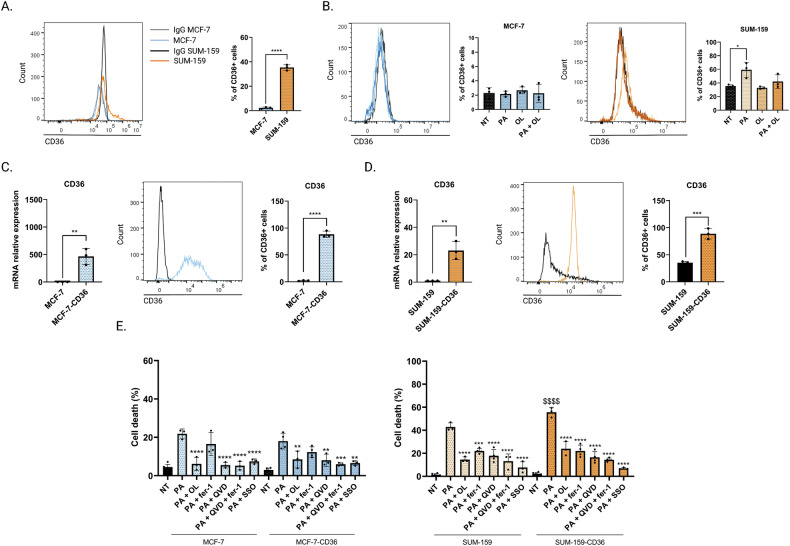
Palmitic acid enhances ferroptosis by upregulating CD36. **A**, **B** CD36 protein expression was assessed by flow cytometry in MCF-7 and SUM-159 cell lines cultured in the absence or in the presence of PA (50 µM), OL (50 µM), or PA (50 µM) combined with OL (50 µM) for 24 h. **C**, **D** Validation by qRT-PCR and by flow cytometry of the overexpression of CD36 in MCF-7 (WT, CD36) and SUM-159 (WT, CD36) cells. **E** Cell death was determined by Annexin-V/7AAD co-labeling in MCF-7 (WT, CD36) and SUM-159 (WT, CD36) cells that were pre-incubated with OL (100 µM, 30 min), fer-1 (5 µM, 6 h), QVD (10 μM, 6 h), or SSO (100 µM, 6 h) and were treated with PA (200 μM, 48 h). The percentages refer to the Annexin-V positive staining. Data are expressed as a mean ± SD (*n* = 3). Data were analyzed by one-way ANOVA; ****p < 0.0001; ***p < 0.001; **p < 0.01; *p < 0.05 vs treated with PA; $$$$*p* < 0.0001 vs SUM-159 WT + PA.

Interestingly, our previous work showed elevated levels of PA in the conditioned media from differentiated adipocytes isolated from breast tumor whereas the level of a monounsaturated FA: oleate (OL) was decreased compared to the normal breast adipose tissue [13]. Moreover, when breast tumor cell lines were incubated with these conditioned media, an increase of mRNA and protein expression levels of CD36 was observed [12]. Therefore, we investigated the expression of CD36 in MCF-7 and SUM-159 cells treated or not with PA, OL, or a combination of PA and OL. At a basal state, MCF-7 cells express low level of the CD36 protein compared to SUM-159 cells, 3% and 35% respectively (Fig. 3A). Interestingly, PA treatment increases CD36 protein expression in SUM-159 but not in MCF-7 cells. OL treatment did not have any effect on CD36 expression. Moreover, the combination of PA with OL abrogated significatively the increased CD36 expression (Fig. 3B and Table S2). Interestingly, another saturated FA, the stearic acid, increases CD36 protein expression (Fig. S4). To further elucidate the potential role of CD36 in PA-induced ferroptosis, we generated efficient stable-transfected cell lines overexpressing the CD36 protein from MCF-7 (MCF-7-CD36) and SUM-159 cells (SUM-159-CD36). The CD36 mRNA and protein overexpression were validated by RT-qPCR and flow cytometry (Fig. 3C, D).

To evaluate the consequences of CD36 overexpression on PA-induced death, the four cell lines were pretreated with QVD, fer-1, OL, and a CD36-specific inhibitor: sulfo-N-succinimidyl oleate (SSO) before PA treatment. Interestingly, SUM-159-CD36 cells exhibited a higher sensitivity towards PA-induced cell death compared to SUM-159 cells. No difference in the cell death response was observed between MCF-7 and MCF-7-CD36 cells. As expected, OL and SSO inhibited PA-related cell death in the four cell lines. QVD but not fer-1 prevented PA-induced death of MCF-7 and MCF-7-CD36 cells. In contrast, fer-1 and QVD were able to abrogate PA-induced cell death of SUM-159 and SUM-159-CD36 cells (Fig. 3E and Table S3).

### CD36 favors uptake of palmitic acid and sensitivity of aggressive breast cancer cells to ferroptosis

**Fig. 4:**
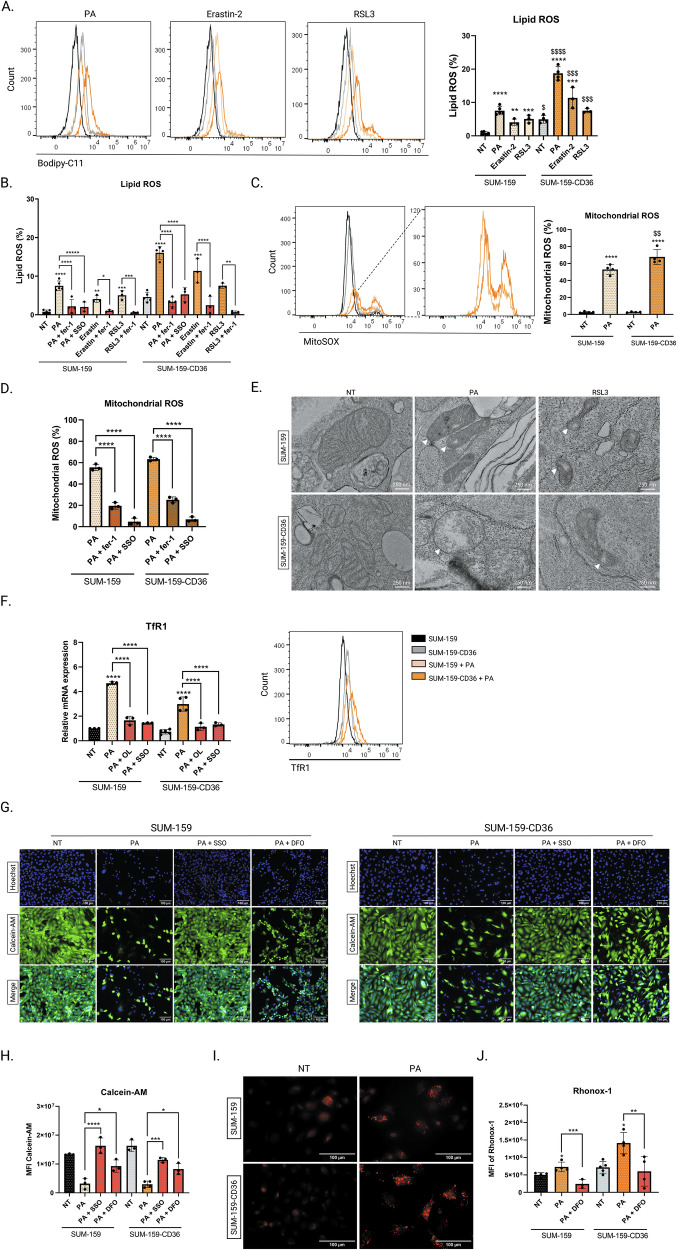
CD36 favors uptake of PA and sensitivity of aggressive breast cancer cells to ferroptosis. **A** SUM-159 (WT, CD36) cells were treated with PA (200 µM), erastin-2 (80 µM), or RSL3 (5 µM) for 48 h and peroxidized lipid production was assessed by Bodipy-C11 labelling (5 µM, 30 min). **B** SUM-159 (WT, CD36) cells were pre-incubated with SSO (100 µM, 6 h) or with fer-1 (5 µM, 6 h) and were treated with PA (200 µM, 48 h), erastin-2 (80 µM, 48 h), or with RSL3 (5 µM, 48 h). The positive cells for the Bodipy-C11 oxidized form of the probe were quantified and expressed as a percentage (*n* = 4). **C**, **D** Mitochondrial ROS over-generation was measured with MitoSOX (5 μM, 30 min) in SUM-159 (WT, CD36) cells that were treated with PA (200 µM, 48 h) alone or combined with fer-1 (5 µM) or SSO (100 µM). **E** TEM of mitochondria in SUM-159 (WT, CD36) cells treated with PA (200 µM, 48 h) or RSL3 (5 μM, 48 h). White arrowheads indicate swollen or damaged mitochondria. Scale bars = 250 nm. **F** mRNA and protein expression level of TfR1 was measured in SUM-159 (WT, CD36) cells pretreated with OL (100 µM, 30 min) or SSO (100 µM, 6 h) before being treated with PA (200 µM, 24 h). **G**, **H** Iron labile pool was measured by calcein-AM (5 µM, 30 min) labelling by immunofluorescence or flow cytometry of SUM-159 (WT, CD36) cells treated with PA (200 µM, 48 h) alone or pre-incubated with SSO (100 µM) or DFO (100 µM) for 6 h before being treated with PA. **I**, **J** Ferrous iron (Fe^2+^) was evaluated by Rhonox-1 staining (5 µM, 30 min) by immunofluorescence and by flow cytometry of SUM-159 (WT, CD36) cells treated with PA (200 µM, 24 h) or **J** pre-incubated with DFO (100 µM, 6 h) or SSO (100 µM, 6 h) before being treated with PA. Data are expressed as a mean ± SD (*n* = 3). Data were analyzed by one-way ANOVA; ****p < 0.0001; ***p < 0.001; **p < 0.01; *p < 0.05 vs non treated; *p* < 0.0001; $$$*p* < 0.001; $$*p* < 0.01; $*p* < 0.05 vs SUM-159 WT.

To investigate the role of CD36 in the PA-induced ferroptosis in TNBC, SUM-159 and SUM-159-CD36 cells were exposed to PA, erastin-2, or RSL3 to evaluate the accumulation of peroxidized lipids. Overexpression of CD36 induced an increase in lipid peroxidation, which could be reversed by fer-1 (Fig. 4A, B). Moreover, SSO prevented PA-induced lipid peroxidation in both SUM-159 and SUM-159-CD36 cells, highlighting the major role of CD36 in the PA-dependent peroxidized lipid accumulation (Fig. 4B). Following PA treatment, CD36 overexpression increased mitochondrial ROS accumulation, which could be abrogated in presence of fer-1 or SSO (Fig. 4C, D). These indicated that CD36 expression is essential in PA-induced mitochondrial ROS accumulation and ferroptosis.

To analyze the effect of CD36 following PA exposure on mitochondrial morphology we performed transmission electron microscopy (TEM). In PA or RSL3 exposed cells, we found swollen mitochondria with decreased or vanished cristae, which are specific characteristics of the ferroptosis death [14, 26]. This was particularly amplified in SUM-159-CD36 cells (Fig. 4E).

Interestingly, we found at protein level but not at mRNA level a higher expression of TfR1 in PA-treated SUM-159-CD36 compared to SUM-159 cells (Fig. 4F). These effects were inhibited in the presence of OL or SSO, supporting the idea that PA-related increased iron uptake is dependent of CD36. According to these results, we found an increased LIP in PA-treated SUM-159-CD36 cells, which was abrogated by SSO and DFO (Fig. 4G, H).

Finally, we found that PA increased the Fe^2+^ content and that CD36 overexpression accentuated this effect, which could be abrogated by DFO (Fig. 4I, J).

**Fig. 5:**
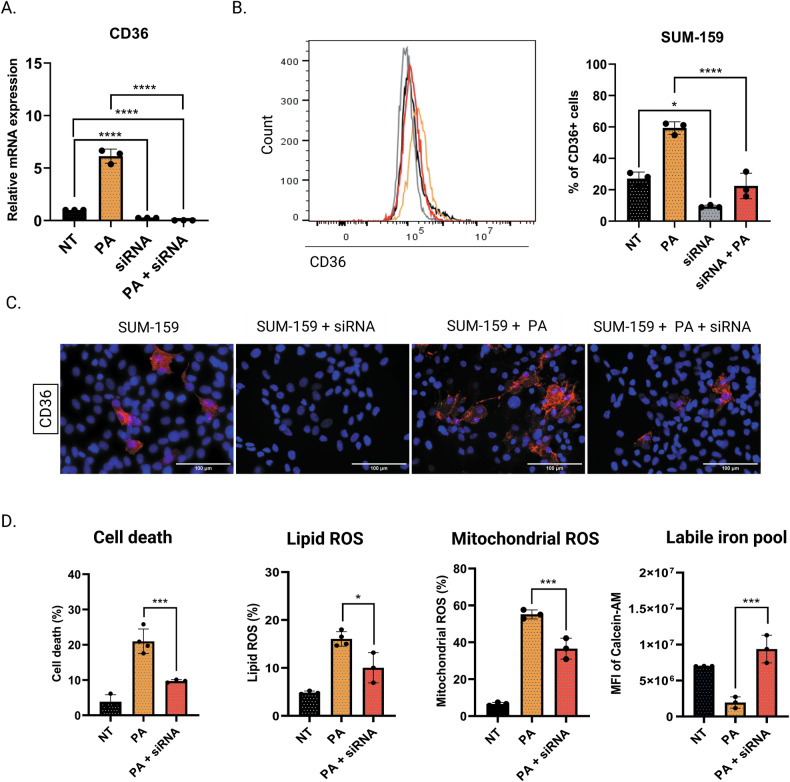
CD36 silencing limits palmitate-induced ferroptosis in SUM-159. **A**–**C** Validation by qRT-PCR, flow cytometry, and by immunofluorescence of the silencing of CD36 in SUM-159 cells pre-treated with 2 µM or with CD36-siRNA for 48 h before being treated or not with PA during 24 h. **D** SUM-159 cells were preincubated with 2 µM of CD36si-RNA for 48 h before being treated or not with PA (200 µM) during 24 h before assessing cell death by Annexin-V/7AAD co-labeling, peroxidized lipid production by Bodipy-C11 labelling (5 µM, 30 min), mitochondrial ROS over-generation with MitoSOX (5 μM, 30 min), and the iron labile pool with calcein-AM (5 µM, 30 min) by flow cytometry. Data are expressed as a mean ± SD (*n* = 3). Data were analyzed by one-way ANOVA; ****p < 0.0001; ***p < 0.001; **p < 0.01; *p < 0.05 vs SUM-159 + PA.

In order to confirm the role of CD36 to palmitate-induced ferroptosis, we silenced CD36 in SUM-159 cells by using two pooled siRNAs and showed a decrease of CD36 expression even in presence of PA at RNA and protein levels (Fig. 5A, B, C). As expected, CD36 silencing limited cell death and prevented lipid peroxidation, mitochondrial ROS production, and increased LIP, confirming the role of CD36 in PA-related ferroptosis (Fig. 5D).

### Expression of CD36 potentiates ferroptosis in TNBC

**Fig. 6:**
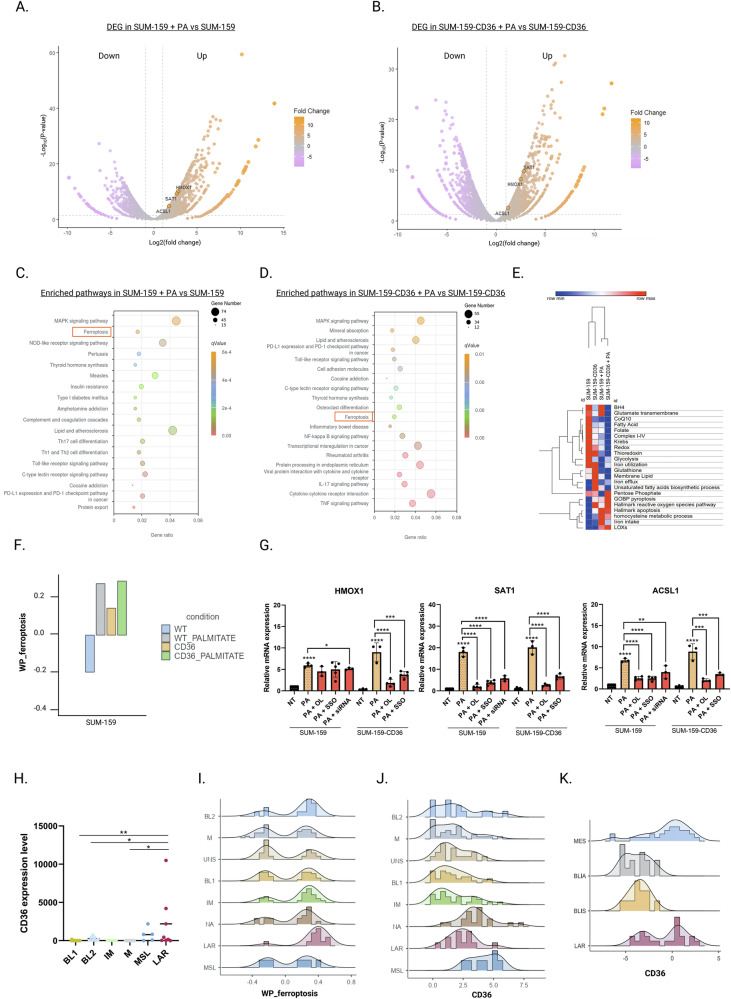
Expression of CD36 potentiates ferroptosis in TNBC. **A** Volcano plot showing the results of differential expression analysis between SUM-159 or (**B**) SUM-159 CD36 cells treated with 200 µM of PA during 24 h vs their non-treated counterparts (orange represents up-regulated expression, violet represents down-regulated expression). The pro-ferroptotic genes HMOX1, SAT1 and ACSL1 are highlighted in orange. **C**, **D** KEGG pathway enrichment analysis of differentially expressed genes showing the altered significant pathways in SUM-159 or SUM-159-CD36 cells following PA treatment. **E** Heatmap showing the normalized ferroptosis-related pathway enrichment scores of SUM-159 (WT, CD36) treated or not with PA (200 µM) during 24 h calculated by single sample gene set enrichment analysis (ssGSEA). **F** Enrichment score of ferroptosis pathway for each sample using gene set variation analysis (GSVA). **G** mRNA expression level of HMOX1, SAT1 and ACSL1 was measured in SUM-159 (WT, CD36) pretreated with OL (100 µM, 30 min), with SSO (100 µM, 6 h) or with 20 nM of CD36si-RNA for 48 h before being treated with PA (200 µM, 24 h). **H** Variations of human CD36 gene expression within the TNBC tumor xenograft (*n* = 54) subtypes classified according to Lehmann classification^22^: basal-like 1 (BL1; *n* = 15), basal-like 2 (BL2; *n* = 7), immunomodulatory (IM; *n* = 4), mesenchymal (M; *n* = 15), unstable (UN), not characterize (NA), mesenchymal stem-like (MSL; *n* = 5) and luminal androgen receptor (LAR; *n* = 8). **I** Enrichment score of ferroptosis pathways among TNBC transcriptomic subtypes according to Lehmann classification in TCGA-TNBC (*n* = 158) cohort. **J** CD36 gene expression within TCGA-TNBC cohort according to Lehmann classification. **K** CD36 gene expression within Jovanović et al. cohort, according to Burstein classification^24^ (*n* = 95) was classified according Burstein classification^24^: the mesenchymal (MES), the basal-like immune activated (BLIA), the basal-like immunosuppressed (BLIS), and the LAR subtypes. Data are expressed as a mean ± SD (*n* = 3). Data were analyzed by one-way ANOVA; ****p < 0.0001; ***p < 0.001; **p < 0.01; *p < 0.05 vs non treated.

To explore the correlation between CD36 and ferroptosis, we performed transcriptomic analysis and evaluated the transcriptomic changes following PA exposition of SUM-159 and SUM-159-CD36 cells (Fig. 6A, B). KEGG (Kyoto Encyclopedia of Genes and Genomes) pathway analysis revealed that upregulated genes due to PA exposure were significantly enriched in ferroptosis pathway, particularly in SUM-159-CD36 cells (Fig. 6C, D). Those results were not found in MCF-7 and MCF-7-CD36 cells treated with PA, as well as SUM-159 and SUM-159-CD36 in absence of PA (Fig. S5, S6A, S6B).

Single-sample gene set enrichment analysis (ssGSEA) was performed to obtain a normalized enrichment score of ferroptosis-related metabolic pathways (Figs. 6E, F and S6C). Interestingly, CD36 overexpression in SUM-159 cells but not in MCF-7 cells increases the enrichment score of the ferroptosis pathway in conjunction with the upregulation of iron utilization, reactive oxygen species pathway, and the downregulation of ferroptosis suppressor pathway, such as CoQ10, thioredoxin, and BH4. This phenomenon was accentuated following PA treatment in both SUM-159 and SUM-159-CD36 cells but not in MCF-7 and MCF-7-CD36 cells. Moreover, the ferroptotic pathway analysis revealed an enrichment of ferroptosis only in SUM-159 and SUM-159-CD36 cells but not in MCF-7 and MCF-7-CD36 cells following PA treatment (Figs. 6F and S6D).

We further validated the increased expression of ferroptotic target genes, especially Heme oxygenase-1 (HMOX1), spermidine/spermine *N*^*1*^-acetyltransferase 1 (SAT1), and acyl-CoA synthetase long-chain family member 1 (ACSL1) following PA treatment (Fig. 6A, G). The combination of PA to OL, to SSO, or to siRNAs targeting CD36 inhibited the upregulated expression of those ferroptosis related-genes (Fig. 6G). We also studied the consequences of PA treatment on the expression of other well-known ferroptosis-related genes and found that PA induced a decreased expression of two key anti-ferroptotic genes: the gluthatione peroxidase 4 (GPX4) and the ferroptosis suppressor protein 1 (FSP1) and an enhanced expression of two key pro-ferroptotic genes: the arachidonate 15-lipoxygenase (ALOX15) and the acyl-CoA synthetase long chain family member 4 (ACSL4). The differential expression due to PA treatment was inhibited by the combination with OL (Fig. S6E).

To further validate the role of CD36 in PA-induced ferroptosis of TNBC, we generated efficient stable-transfected cell line overexpressing the CD36 protein from another TNBC cell line: the MDA-MB-231 (MDA-MB-231-CD36). The CD36 mRNA and protein overexpression were validated by RT-qPCR and flow cytometry (Fig. S7A). We found that CD36 overexpression increases MDA-MB-231 sensitivity towards PA (Fig. S7B). We then evaluated the expression of the previously identified ferroptotic related genes HMOX1, SAT1, and ACSL1. We found similar results in MDA-MB-231 overexpressing CD36 as in SUM-159, except for ACSL1, which did not have its expression impacted by PA treatment (Fig. S7C).

### LAR is a TNBC subtype sensitive to CD36-induced ferroptosis

We finally evaluated the expression of CD36 in a large cohort of patient-derived xenograft (PDX) derived from breast cancer patients classified in six different triple negative subgroups according to Lehmann classification [27] (Fig. 6H). We found that CD36 was particularly expressed in the PDX coming from TNBC of the LAR subtype, which is known to be more sensitive to ferroptosis [22]. Thus, we validated these observations in patient databases and chose to use the transcriptomic data of two cohorts coming from the TCGA and from Jovanović et al. [28] (Fig. 6I–K). Jovanović’s cohort was classified according Burstein classification [29]. We validated an enrichment of ferroptosis in the LAR subtype (Fig. 6I). Concomitantly, we found CD36 particularly expressed on this subtype in both cohorts, validating its role in the ferroptotic process in TNBC (Fig. 6J, K).

## Discussion

Breast cancer is the most prevalent cancer among women [1]. Thus, identifying new predictive and prognostic markers as well as new therapeutic targets in order to effectively counteract the development of severe forms of this cancer is mandatory. It will help reduce the adverse effects induced by traditional anti-cancer treatments such as chemotherapy.

Here, through in vitro studies, patient-derived xenografts, and patient databases, we identified CD36 as a potentially interesting marker for the sensitivity of TNBC cells to PA-induced ferroptosis.

CD36 is well-known for its role in free FA transport in all cells with a metabolic role. In cancer, CD36 is found to be overexpressed at a metastatic state in numerous carcinomas, including breast cancer [4, 8, 30]. CD36 appears to play an ambivalent role in tumor progression with pro- and anti-tumoral roles [6]. Indeed, on one hand, CD36 promotes ferroptosis in key anti-tumor immunity cells, CD8 + T lymphocytes, by increasing their uptake of polyunsaturated fatty acids (PUFAs), thereby altering their cytokine production and anti-tumor activity, thus promoting tumor progression [31]. On the other hand, it was shown in a colon cancer model that CD36, by favoring the uptake of PA, directly promotes the sensitivity of the cancer cells themselves to ferroptosis [32].

Interestingly, recent data have demonstrated the importance of FA availability and composition of the tumor microenvironment in the ability of cancer cells to execute the ferroptotic program [33]. Indeed, many studies have pointed out the role of PUFAs, which are sensitive to peroxidation, and the counteracting role of monounsaturated FAs, particularly OL, in the limitation of this peroxidation [33–35]. The increased level of those PUFAs could be due on one hand, to an increased biosynthesis linked to a high expression of the fatty acid desaturases 1 and 2 (FADS1/2) as shown in TNBC [36]. On the other hand, PUFA level can also be influenced by an increased uptake of exogenous FA due to an increased expression of FA transporter such as CD36 [12]. Nevertheless, recent studies have shown that not only PUFAs but saturated FAs, especially PA, could be involved in ferroptosis. Indeed, Guo et al. also highlighted the ability of PA to induce ferroptosis through ceramide synthesis and the inhibition of GPX4, a master regulator of ferroptosis [37]. Concomitantly, recent studies have highlighted the ability of PA to induce ferroptosis in numerous cell types, such as cardiomyocytes [38], pancreatic β-cells [37], and even colon cancer cells [32].

However, this question has not yet been addressed in breast cancer. Thus, we studied the possible role of PA in the induction of ferroptosis in breast cancer cells through CD36. Our data showed that luminal breast cancer cells were less sensitive towards PA compared to TNBC cells, which could be explained at least in part by their differential uptake of this FA. Although PA only induced apoptosis in luminal cells, it triggers both apoptosis and ferroptosis in TNBC cells. This was consistent with a study highlighting that estrogen receptor-positive breast cancer cells, such as luminal A cancer cells, are resistant to ferroptosis [39]. This resistance is due to the upregulation of the subunits (SLC7A11 and SLC3A2) of the cystine/glutamate antiporter system x_c_- which imports cystine in exchange for glutamate, sustaining the production of glutathione and thus the function of GPX4 in those cells. Interestingly, estrogens, especially 17β-estradiol, have been recently shown to favor a resistance towards ferroptotic death through both genomic and non-genomic mechanisms [40].

Furthermore, the sensitivity of TNBC towards those two types of cell death demonstrates the presence of diversified subpopulations of TNBC cells that could undergo different types of cell death. Recent evidence also indicates a possible crosstalk between apoptosis and ferroptosis, especially through the induction of ER stress [41, 42].

We also found that SSO significantly inhibited cell death following PA treatment in SUM-159 and MCF-7 cells, confirming the involvement of CD36 in PA-induced apoptosis and ferroptosis in our models. We also confirmed those results by using CD36-siRNA on SUM-159. Collectively, these results suggest that CD36 expression could explain the sensitivity of SUM-159 towards PA-induced ferroptosis in contrast to MCF-7.

Our previous work has shown that TNBC cell lines exposed to conditioned media coming from adipocytes overexpressed CD36 [12]. We also showed that the secretome of differentiated adipocytes coming from tumor-bearing women was more enriched in PA and decreased in OL compared to the secretome coming from tumor-free breast adipose tissue [13]. Then, to uncover the specific role of CD36 in breast cancer cells' sensitivity towards ferroptosis, we overexpressed CD36 in luminal A and TNBC cells. This increased expression was not sufficient to induce ferroptosis in luminal A breast cancer cells. This could also be explained, in part by the fact that luminal A breast cancer cells, unlike our model of TNBC, are lacking expression of CD44, which has been shown to be the main effector of iron uptake, a key mechanism in the induction of ferroptosis [23, 43]. While this overexpression did increase the ferroptotic features in the TNBC cells, especially the lipid peroxidation. This higher peroxidized lipid level was also observed following erastin-2 and RLS3-induced ferroptosis in TNBC overexpressing CD36 cells, suggesting that CD36 itself favors the ferroptotic process independently of PA. In line with our results, a recent study has shown the ability of CD36 to regulate a glutathione-independent ferroptosis suppressor: ferroptosis suppressor protein 1 (FSP1) ubiquitination, thus favoring its degradation and inducing the ferroptosis process [44]. Concomitantly, another recent study has shown that a PARP inhibitor could limit the formation of omental metastasis from ovarian cancer cells through the upregulation of CD36, leading to ferroptosis induction [45].

We then found that PA treatment combined with CD36 overexpression increases mitochondrial ROS production, TfR1 protein expression, and the iron content of TNBC cells overexpressing CD36 compared to the wild-type TNBC. This highlighted a specific role of the fatty acid transporter CD36 in the induction of PA-induced ferroptosis in TNBC.

To better unravel the molecular mechanisms leading to ferroptosis following CD36 overexpression, we performed transcriptomic analyses and therefore identified ferroptosis as an enriched pathway, especially in CD36-overexpressing TNBC following PA treatment. In addition, our findings demonstrated the activation of the iron uptake and ROS pathways. We specifically identified three genes implicated in ferroptosis and enriched in our models: HMOX1, SAT1, and ACSL1.

A recent study has described the role of HMOX1 in ferroptosis due to its function of heme decomposition leading to Fe^2+^ release [46]. This is consistent with our previous results showing an increased iron pool in CD36-overexpressing TNBC cells following PA treatment. Moreover, SAT1 has been demonstrated as an inducer of arachidonate 15-lipoxygenase (ALOX15) expression, a key enzyme involved in lipid peroxidation of PUFAs, especially the arachidonic acid [47]. Finally, we identified ACSL1, which has been shown to favor ferroptosis through the incorporation of PUFAs, especially conjugated linoleates, into triacylglycerols, favoring their oxidation after exceeding a certain PUFA level [48]. We validated the increased expression of HMOX1 and SAT1 but not ACSL1 genes following PA treatment in another TNBC cell line overexpressing CD36 (MDA-MB-231-CD36), indicating the importance of these two genes in the ferroptosis process induced by CD36.

Finally, a recent report has shown that TNBCs have heterogeneous ferroptosis phenotypes according to their transcriptomic-base subtypes. Indeed, the authors showed that the TNBC LAR subtype, characterized by high expression of the androgen receptor, an increased ROS accumulation, and FA synthesis making LAR tumors highly sensitive to ferroptosis [22]. Interestingly, we found CD36 expressed at higher level in LAR subtype-patients derived-xenograft compared to the PDX coming from other TNBC subtypes, and we validated those observations using patients’ data coming from two databases.

We also found higher CD36 expression, but to a lesser extent than for LAR subtype, in mesenchymal-like subtype of TNBC (MSL and MES) also shown to be sensitive to ferroptosis due to their high iron uptake [22], validating the correlation between CD36 expression and ferroptosis.

All together, these data suggest a crucial role of CD36 in PA-induced ferroptotic cell death in TNBC through the increase of different hallmarks of ferroptosis, especially peroxidized lipid, mitochondrial ROS accumulation, and mitochondria morphology, as well as increased iron uptake leading to an increase Fe^2+^ content. Thus, CD36 could be a marker of TNBC cells with a high susceptibility towards ferroptosis, as illustrated here by the PA induction of this cell death. This could open new therapeutic strategies, increasing cancer cells' sensitivity to ferroptosis in breast cancer patients who are refractory to other anti-cancer therapies with high expression of CD36.

## Materials and methods

### Cell culture

MCF-7(#HTB-22), ZR-75.1 (#CRL-1500), and MDA-MB-231 (#HTB-26) cells obtained from American Type Culture Collection (ATCC, LGC Standards, Molsheim, France) were maintained in DMEM medium (Dulbecco modified Eagle’s medium) supplemented with 10% (*v/v*) FBS (fetal bovine serum). The SUM159PT cell line (provided by Philippe Benaroch, Cellular transport and immunity team, Curie Institute, Paris, France) was maintained in Ham’s F12 medium supplemented with 5% heat inactivated FBS, 10 mM HEPES, 1 µg/mL hydrocortisone, and 5 µg/mL of human insulin. The T47D (#HTB-133) and BT549 (#HTB-122) cell lines obtained from ATCC were maintained in RPMI 1640 medium supplemented with 10% (*v/v*) FBS (fetal bovine serum). Cell lines were regularly checked for mycoplasma contamination. All the cultures were maintained at 37 °C in a humidified incubator with 5% CO_2._

### Cell Transfection

Cells were transfected with the plasmid encoding CD36 (Sino Biological, Beijing, China) using the Lipofectamine 3000 reagent (Thermo Fisher Scientific, Waltham, Massachusetts, USA) for 48 h. Then, cells were placed in a selective medium containing hygromycin B (100 µg/mL for MCF-7 and 200 µg/mL for SUM-159 and MDA-MB-231). After selection, the drug-resistant cells were cloned, harvested, and stored.

### Transient siRNA transfection

SUM-159 cells were seeded into 12-well plates (1 × 10^5^ per well) in corresponding growth media containing 5% FBS to achieve 70% confluence. The following day, cells were transfected with 20 nM of siRNA targeting CD36 (sc-29995) from Santa Cruz Biotechnology (Santa Cruz Biotechnology, Dallas, Texas, USA) using Lipofectamine RNAiMAX Reagent from Thermo Fisher Scientific in Opti-MEM media from Gibco accordingly to manufacturer’s instructions for 48 h, refreshed every day before being treated or not with PA (200 µM) during 24 h. Control cells were treated with transfection reagent only. The functional analyses were performed 72 h after transfection.

### Cell viability assay

Cell viability was assessed by CellTiter Glo Luminescent Viability assay 2.0 (Promega, Madison, Wisconsin, USA). Briefly, 7 × 10^3^ cells seeded in a white 96-well plate in medium supplemented with 2% FBS, were treated with various concentrations of PA. After 24 h, 48 h, or 72 h of treatment, a CellTiter Glo assay was performed as recommended by Promega. Luminescence was measured in a microplate reader, Infinite 200Pro (Tecan^®^, Austria). All values are averages of at least three independent experiments done in duplicate. The IC_50_ values were calculated with the Excel software.

### Antibodies and reagent

Bodipy FL-C12 (D3822), Bodipy^TM^ 581/591 C11 (D3861), MitoSOX^TM^ Red (M36008), and Calcein-AM (C3100MP) were purchased from Thermo Fisher Scientific. Palmitic acid (P-9767), Ferrostatin-1 (SML0583), Erastin-2 (SML2794), OL (01383), QVD-Oph (SML0063), Necrostatin-1 (N9037), and Deferoxamine (DFO, D9533) were purchased from Sigma-Aldrich-Merck (Saint-Louis, MO, USA). RSL3 (S8155) was purchased from Selleckchem (Boston, Massachusetts, USA) and SSO (ab145039) from Abcam (Cambridge, UK). The following antibodies or probes were used: PE Annexin V Apoptosis Detection Kit with 7-AAD (640934, BioLegend, San Diego, California, USA), TfR1 (IM2001U, Beckman, Coulter, Brea, California, USA), CD36 (5114-PE100T, BioCytex, Marseille, France), HMRhoNox-M, Fe(II) (3317, Luminoprobe, Hunt Valley, Maryland, USA).

### Cell death induction and inhibition

Cells seeded at 1 × 10^5^ per well in a 12-well plate with medium supplemented with 2% FBS were pre-treated with the inhibitors QVD-Oph (pan-caspases inhibitor, 10 µM), Necrostatin-1 (RIPK1 inhibitor, 40 µM), Ferrostatin-1 (ferroptosis inhibitor, 5 µM), Sulfo-N-succinimidyl Oleate sodium (CD36 inhibitor, 100 µM). After 6 h exposure, cells were treated with PA (200 µM) combined or not with OL (100 µM) for 48 h. Cells were then washed with PBS, harvested with Accutase (Merck), and collected together with the cell culture medium. Cells were then double-stained with Annexin-V-PE and 7-amino-actinomycin D (7AAD) (PE Annexin V Apoptosis Detection Kit with 7-AAD; BioLegend). Cell death induction was recorded for the total population in a cytoFLEX (Beckman Coulter), and data were analyzed using FlowJo software.

### Lipid peroxidation assessment

Peroxidized lipid level was detected by determined using BODIPY^TM^ 581/591 C11 (Invitrogen) staining. For flow cytometry, cells were seeded at 1 × 10^5^ per well in a 12-well plate with medium supplemented with 2% FBS during 24 h before being treated as indicated. Then, cells were washed with PBS and detached with Accutase before being resuspended in PBS with 0.5% bovine serum albumin (BSA) and incubated with C11-BODIPY (5 µM) for 30 min with 5% CO_2_ at 37 °C. Fluorescence was assessed using a cytoFLEX cytometer (Beckman Coulter). For immunofluorescence staining, cells were rinsed three times with PBS before being incubated with BODIPY^TM^ 581/591 C11 dye in serum-free medium at 37 °C with 5% CO2 for 30 min before being visualized under a fluorescence microscope (Evos FL, Thermo Fischer Scientific) with objective ×40.

### Mitochondrial ROS measurement

Mitochondrial ROS level was determined using mitochondrial ROS sensor MitoSOX^TM^ Red (Invitrogen) staining. Cells were seeded at 1 × 10^5^ per well in a 12-well plate with medium supplemented with 2% FBS during 24 h. After being exposed to different treatments, cells were washed with PBS and detached with Accutase before being resuspended in PBS with 0.5% BSA. Then, cells were incubated with 5 µM of MitoSOX^TM^ Red dye in PBS with 0.5% serum at 37 °C with 5% CO_2_ for 30 min before being assessed in a cytoFLEX cytometer (Beckman Coulter).

### Flow cytometry analysis

For cell surface staining, cells cultured in medium supplemented with 2% serum and treated as indicated were detached with Accutase treatment and re-suspended in PBS supplemented with 0.5% serum. Cells were then incubated for 20 min at room temperature in the dark with the relevant antibody (CD36 or TfR1). Cells were then washed and suspended in PBS containing 0.5% serum prior to being analyzed by flow cytometry.

For each condition, at least 10,000 events were counted per sample. Data were recorded in a cytoFLEX cytometer (Beckman Coulter) and analyzed using FlowJo software.

### Immunofluorescence

SUM-159 cells were first rinsed three times with PBS. Next, they were fixed in 4% paraformaldehyde (PFA) for 30 min at room temperature. After fixation, cells were permeabilized with 0.1% Triton X-100 for 15 min, followed by blocking with 1% BSA for 30 min at room temperature. Following washing with PBS, the primary antibody, CD36 (5114-PE100T, 1:20 dilution, BioCytex), was added, and cells were incubated at 4 °C overnight. The next day, cells were washed three times with PBS, then were incubated with 4′,6′-diamidino-2-phenylindole (DAPI, 1 µg/mL) during 5 min at RT. Then slides were mounted with and visualized under a fluorescence microscope (Evos FL, Thermo Fischer Scientific) with objective ×40.

### Transmission electron microscopy (TEM)

Cells were seeded in 60 mm cell culture dishes (Corning, NY, USA) and treated with PA (200 µM) or RSL3 (5 µM) for 48 h. Then, samples were first fixed with 50% of culture medium and 50% of 2% PFA with 2% of glutaraldehyde Hepes buffer 0.1 M at pH 7.4 for 1 h before being fixed with 2% of paraformaldehyde (PFA) with 2% of glutaraldehyde Hepes buffer 0.1 M for one night at 4 °C. Then cells were washed with cacodylate buffer 0.1 M and then post-fixed with 1% osmium tetroxide in water for 1 h at RT. After dehydration and embedding in epoxy resin, blocks were cut with a UC7 ultramicrotome (Leica, Leica Microsystems SAS, Nanterre, France). Ultrathin sections were observed with a Hitachi HT7700 electron microscope (Milexia, Saint Aubin 91190, France) operating at 100 kV. Pictures (4096 × 4096 pixels) were taken with a Nanosprint12 camera. TEM was performed by the ICM quant facility (Paris Brain Institute).

### Iron dosage

For immunofluorescence staining, cells were plated and grown in the appropriate medium supplemented with 2% FBS 24 h before being treated as indicated. Live cells were then incubated for 1 h in medium containing Calcein-AM (2 µM) and Hoechst or RhoNox-M (5 µM) at 37 °C with 5% CO_2_. Then, cells were subsequently washed three times with PBS, and images were acquired using an EVOS microscope (Thermo Fisher Scientific).

For flow cytometry experiments, cells were prepared as indicated in the flow cytometry section and incubated for 30 min at 37 °C with Calcein-AM (10 µM) or Rhonox (5 µM) before being washed twice with PBS and suspended in PBS containing 0.5% serum prior to being analyzed.

### Quantitative real-time RT-PCR

The RNA was extracted by TRIzol reagent (ThermoFisher Scientific) from cells treated or not with 200 µM of PA during 24 h. Total RNA was extracted using the RNeasy^®^ Plus Micro Kit (Qiagen, Hilden, Germany). The RNA quantity and purity were determined using a Spectrophotometer DS-11 (Denovix, Wilmington, DE, USA). One microgram of total RNA from each sample was reverse transcribed, and the real-time RT-PCR measurements were performed, as described previously [49], using an apparatus Aria MX (Agilent Technologies, Santa Clara, CA, USA) with the corresponding SYBR^®^ Green kit, according to the Promega manufacturer’s recommendations. The data were analyzed using the comparative threshold cycle 2^−ΔΔCt^ method; the expression of 36B4 (RPL0) and β-actin was used to normalize the data (Table S1). The expression of the genes was expressed as the fold change in the samples, compared to the control condition. The gene specific primers were purchased from Sigma Aldrich-Merck and are listed in supplemental Table 1.

### RNA sequencing

RNA was extracted by TRIzol reagent (Thermo Fisher Scientific) from cells treated or not with PA (200 µM, 24 h). RNA integrity (RIN) was assessed using the RNA 6000 Nano kit (Agilent) and a Bioanalyzer 2100 system (Agilent). PolyA mRNA selection, cDNA libraries preparation, and sequencing were conducted by Novogene (Munich, Germany) using the Illumina NOVAseq X Plus platform. Differential expression analysis was performed using the DESeq2 R package (1.20.0). The resulting p-values were adjusted using Benjamini and Hochberg’s approach for controlling the false discovery rate. Genes with an adjusted *p*-value ⩽ 0.05 and a |log2(FoldChange)| ≥ 2 found by DESeq2 were assigned as differentially expressed. Gene Ontology (GO) enrichment analysis of differentially expressed genes was implemented by the cluster Profiler R package, in which gene length bias was corrected. GO terms with corrected *p*-value less than 0.05 were considered significantly enriched by differential expressed genes. Cluster Profiler R package has been used to test the statistical enrichment of differential expression genes in KEGG pathways. The figures were designed performed using NovoMagic, a free Novogene platform for data analysis. RNAseq data are deposited on the NCBI under accession number GSE306759.

### Patient-derived xenografts

Patient-derived xenografts (PDXs) of TNBC have been established from 54 patient breast tumors as previously described [50–52]. The TNBC molecular subtype according to the Lehman classification [27] was determined from gene expression data as detailed by Chen et al. [53]: basal-like 1 (BL1; *n* = 15), basal-like 2 (BL2; *n* = 7), immunomodulatory (IM; *n* = 4), mesenchymal (M; 4 *n* = 15), unstable (UN), not characterize (NA), mesenchymal stem-like (MSL; *n* = 5) and luminal androgen receptor (LAR; *n* = 8). Briefly, breast cancer fragments were obtained from patients conventionally treated by conserving surgery or mastectomy at the Institut Curie hospital, with their informed consent, between 2003 and 2023. Tumour fragments of 30–60 mm^3^ were grafted into the interscapular fat pad of 8- to 12-week-old female Swiss Nude mice Crl:NU(Ico)-Foxn1nu or CB17/ Icr-Prkdcscid/IcrCrl42-45 purchased from Charles River Laboratories (Les Arbresles, France). Mice were maintained in specific pathogen-free animal housing. Xenografts appeared at the graft site 2–8 months after grafting. They were routinely passaged by subcutaneous engraftment in Swiss Nude mice in accordance with national regulation and international guidelines [50, 54] and the rules of the French Ethics Committee: CEEA-IC (Comité d’Ethique en matière d’expérimentation animale de l’Institut Curie, National registration number: #118). (Project authorization no. 02163.02).

### Triple-negative breast cancer (TNBC) datasets

We queried the TCGA database in order to retrieve clinical as well as gene expression profiles of 158 TNBC. Clinical data and normalized read counts generated from RNA-sequencing were downloaded using cBioPortal [55, 56] and the TCGA2STAT R-package, respectively [57]. We retrieved for each sample from a previous publication [58] the corresponding molecular subtype as previously defined. We retrieved subtypes annotation and the level of CD36 gene expression in 95 TNBC from a previous cohort DFCI of patients included in a clinical trial at Dana Farber Cancer Institute [28].

### Bioinformatic analysis

Bioinformatics analyses were performed using Bioconductor packages in the R language [59] as well as the Jamovi (The jamovi project (2024). jamovi (Version 2.5) [Computer Software]. Retrieved from https://www.jamovi.org). Enrichment scores of previously published biological pathways related to ferroptosis [22, 60] were computed using the Gene Set Variation Analysis (GSVA) R package. The GSVA R package is a non-parametric unsupervised method for assessing gene set enrichment in gene expression microarray and RNA-seq data [61]. Unlike other methods that analyse differential pathways between two phenotypical groups, the GSVA tool allows for computing an enrichment score (ES) of a given gene set in each sample, with a bimodal distribution of ES. Default parameters were used (abs.ranking = false, tau = 1). This method, based on the Kolmogorov-Smirnov (KS) like random walk statistic, allows to produce a bimodal distribution of enrichment scores by generating non-zero maximum deviations under the null distribution. Contrary to alternative method based on a ‘competitive’ hypothesis, GSVA is based on ‘self-contained null hypothesis’ which analyzes each gene set in isolation, assessing differential expression of the gene set without comparing to a background [62, 63]. As described by the authors, the main strength of GSVA lies in its capabilities for analyzing single samples. Hierarchical clustering was performed using the Morpheus Software (https://software.broadinstitute.org/morpheus), with the Pearson correlation and average linkage method.

### Statistical analysis

Data were analyzed using one-way ANOVA test. Data are presented as means ± SD of three independent experiments. *p* values < 0.05 were considered significant.

## Supplementary information

Supplementary figure legends

Supplementary tables

FIGURE S1

FIGURE S2

FIGURE S3

FIGURE S4

FIGURE S5

FIGURE S6

FIGURE S7

## Data Availability

The RNA-sequencing data supporting this study have been deposited at the NCBI under accession number GSE306759.
