# Supplementary material for: CD36 enhances sensitivity of triple negative breast cancer cells to palmitate-induced ferroptosis
Source: Cell Death Dis. 2026 Feb 11;17(1):219. doi: 10.1038/s41419-026-08460-3 (PMC12920903; doi:10.1038/s41419-026-08460-3)
Supplement: Supplementary file 1 — Supplementary figure legends [file 41419_2026_8460_MOESM1_ESM.pdf]

Supplementary Figure Legends

Figure S1. PA induces cell death in MCF-7 and SUM-159 cells. Cell death was determined by Annexin-V/7AAD co-labeling in MCF-7 and SUM-159 cells that were treated with PA (200 µM) for 48 h. The Annexin-V/7AAD graph is representative of three independent experiments.

Figure S2. Palmitic acid promotes cell death in different subtypes of breast cancer cells. (A) ZR-75.1, T47D, BT549 and MDA-MB-231 cells were treated with increasing doses of PA (300, 500, 1000 and 2000 µM for ZR-75.1) and (20, 30, 50 and 100 µM for the three other cell lines) during 24 h, 48 h and 72 h. Cell viability was assessed by using ATP-based CellTiter-Glo ® 2.0 assay. (B) Cell death was determined by Annexin-V/7AAD co-labeling in ZR-75.1, T47D, BT549 and MDA-MB-231 cells that were pre-incubated with fer-1 (5 µM), QVD (10 µM) or necro-1 (40 µM) for 6 h and then were treated with PA (2 mM, 50 µM, 20 µM and 70 µM respectively) for 48 h. Data are expressed as a mean ± SD (n=3). Data were analyzed by one-way ANOVA; ***$p < 0.001$; **$p < 0.01$; < 0.05 vs non treated.

Figure S3. Ferrostatin-1 does not inhibit PA induced mitochondrial ROS in MCF-7.
Mitochondrial ROS was measured with MitoSOX (5 µM, 30 min) in MCF-7 cells that were pre-incubated or not with fer-1 (5 µM, 6 h) before being treated with PA (200 µM, 48 h). Data are expressed as a mean ± SD (n=3). Data were analyzed by one way ANOVA ; ***$p < 0.001$; **$p < 0.01$; < 0.05 vs non treated.

Figure S4. Stearic acid increases CD36 expression.
CD36 protein expression was assessed by flow cytometry in SUM-159 cell lines cultured in the absence or in the presence of stearic acid (50 µM) for 24 h. Data are expressed as a mean ± SD (n=3). Data were analyzed by student t-test ; ****$p < 0.0001$ vs non treated.

Figure S5. Influence of CD36 overexpression on SUM-159 signaling pathways. KEGG pathway enrichment analysis of differentially expressed genes showing the significant pathways that had been altered in SUM-159 or SUM-159 CD36 cells following or not PA treatment (200 µM, 24 h).

Figure S6. CD36 overexpression does not impact the ferroptosis pathway in MCF-7 following PA treatment. (A) KEGG pathway enrichment analysis of differentially expressed genes showing the significant pathways that were altered in MCF-7 or MCF-7-CD36 cells following PA treatment (200 µM, 24 h). (B) Heatmap showing the normalized ferroptosis-related pathway enrichment scores of MCF-7 (WT, CD36) cells and SUM-159 (WT, CD36) cells treated or not with PA (200 µM) during 24 h calculated by ssGSEA. (C) Enrichment score of ferroptosis pathways for each sample calculated by ssGSEA. (D) mRNA expression level of GPX4, FSP1, ALOX15 and ACSL4 was measured in SUM-159 (WT, CD36) cells that were pre-incubated or not with OL (100 µM, 30 min) before being treated with PA (200 µM, 24 h). Data are expressed as a mean ± SD (n=3). Data were analyzed by one-way ANOVA; ***$p < 0.001$; **$p < 0.01$; < 0.05 vs non treated.

Figure S7. CD36 overexpression increases PA sensitivity and ferroptosis-related genes expression in MDA-MB-231. (A) Validation by qRT-PCR and by flow cytometry of the overexpression of CD36 in MDA-MB-231 (WT, CD36). (B) MDA-MB-231 (WT, CD36) cells were treated with increasing doses of PA (10, 20, 30, 50 and 100 µM) during 48 h. Cell viability was assessed by using ATP-based CellTiter-Glo ® 2.0 assay. (C) mRNA expression level of HMOX1, SAT1 and ACSL1 was measured in MDA-MB-231 (WT, CD36) pretreated with OL (35 µM, 30 min) or SSO (100µM, 6 h) before being treated with PA (70 µM, 24 h). Data are expressed as a mean ± SD (n=3). Data were analyzed by one-way ANOVA; ***$p < 0.001$; **$p < 0.01$; < 0.05 vs non treated.
