# Supplementary material for: CD36 enhances sensitivity of triple negative breast cancer cells to palmitate-induced ferroptosis
Source: Cell Death Dis. 2026 Feb 11;17(1):219. doi: 10.1038/s41419-026-08460-3 (PMC12920903; doi:10.1038/s41419-026-08460-3)
Supplement: Supplementary file 2 — Supplementary tables [file 41419_2026_8460_MOESM2_ESM.pdf]

Supplementary Tables

Table S1. List of primers used in the present study.

Gene	Foward	Reverse
**CD36**	TGGTACAGATGCAGCCTCAT	AGGCCTTGGATGGAAGAACA
**TfR1**	AAAATCCGGTGTAGGCACAG	TTAAATGCAGGGACGAAAGG
**HMOX1**	TCCGATGGGTCCTTACACTC	TAAGGAAGCCAGCCAAGAGA
**SAT1**	CCGTGGATTGGCAAGTTATT	TCCAACCCTCTTCACTGGAC
**ACSL1**	CCAGAAGGGCTTCAAGACTG	GCCTTCTCTGGCTTGTCAAC
**GPX4**	GCCAGGGAGTAACGAAGAGA	CAGCCGTTCTTGTCGATGAG
**FSP1**	TGTGTGTGTACGTGCTTGTG	TGATGGCAGAGATTTGGGGT
**ALOX15**	GTGGAAAACAGTGTGGCCAT	AGTAAGGTCCCAGGTGATGC
**ACSL4**	GGTGGTTTTGCACTGGTGAT	CCCCTTTCTGTTGTGCCAAA
**βActine**	GGACTTCG AGCAAGAGATGG	AGCACTGTGTTGGCGTACAG
**36B4**	GGACTTCG AGCAAGAGATGG	AGCACTGTGTTGGCGTACAG

Table S2. Ajdusted p-values of figure 3B.

Cell line	Condition	Adjusted P-value
MCF-7	NT vs PA	0.9971
	NT vs OL	0.9394
	NT vs PA + OL	>0.9999
SUM-159	NT vs PA	0.0131
	NT vs OL	0.9595
	NT vs PA + OL	0.6070

Table S3. Adjusted p-values of figure 3E.

Cell line	Condition	Adjusted P-value
MCF-7	PA vs PA + OL	<0.0001
	PA vs PA + fer-1	0.1381
	PA vs PA + QVD	<0.0001
	PA vs PA + QVD + fer-1	<0.0001
	PA vs PA + SSO	<0.0001
MCF-7-CD36	PA vs PA + OL	0.0057
	PA vs PA + fer-1	0.0830
	PA vs PA + QVD	0.0041
	PA vs PA + QVD + fer-1	0.0008
	PA vs PA + SSO	0.0013
SUM-159	PA vs PA + OL	<0.0001
	PA vs PA + fer-1	0.0001
	PA vs PA + QVD	<0.0001
	PA vs PA + QVD + fer-1	<0.0001
	PA vs PA + SSO	<0.0001
SUM-159-CD36	PA vs PA + OL	<0.0001
	PA vs PA + fer-1	<0.0001
	PA vs PA + QVD	<0.0001
	PA vs PA + QVD + fer-1	<0.0001
	PA vs PA + SSO	<0.0001
SUM-159 vs SUM-159-CD36	PA vs PA	0.017
